# Mirizzi Syndrome with Cholecystobiliary Fistula: Observation of Development from Asymptomatic Cholecystolithiasis to Surgery

**DOI:** 10.1155/2020/2049525

**Published:** 2020-01-27

**Authors:** Hiroyuki Sugo, Yuuki Sekine, Naoki Iwanaga, Shigefumi Neshime, Michio Machida

**Affiliations:** Department of General Surgery, Juntendo University Nerima Hospital, 3-1-10 Takanodai, Nerima-ku, Tokyo 177-8521, Japan

## Abstract

Despite a considerable number of reports of Mirizzi syndrome, none have described the process of its development from simple cholecystolithiasis. We report an extremely rare case of Mirizzi syndrome in which it was possible to observe the process of development of cholecystobiliary fistula from asymptomatic cholecystolithiasis until unavoidable surgical intervention 4 years later. A 68-year-old woman presented at our hospital with right upper quadrant pain. She had been diagnosed as having asymptomatic cholecystolithiasis 4 years previously. Diagnostic abdominal computed tomography (CT) had revealed a 1.9 cm radiopaque stone, and thereafter, the patient had been monitored by imaging alone. CT conducted 6 months before the present admission revealed that the gallbladder stone was compressing the common hepatic duct, although the patient remained asymptomatic. On admission, abdominal CT showed that the gallbladder stone was obstructing the common bile duct with dilatation of the intrahepatic duct. Endoscopic retrograde cholangiopancreatography revealed a round filling defect at the confluence of the common bile duct and the image of the cystic duct; therefore, the patient was categorized as having Mirizzi syndrome type III, according to the Csendes classification. Intraoperative findings revealed a cholecystobiliary fistula involving up to two-thirds of the circumference of the common bile duct.

## 1. Introduction

Mirizzi syndrome is characterized by symptoms of recurrent biliary colic, obstructive jaundice, or cholangitis, which are caused by bile duct obstruction due to a stone impacted in the Hartmann pouch or cystic duct. The reported incidence ranges from 1% to 2% of patients with symptomatic cholelithiasis [1]. Although the incidence of Mirizzi syndrome is low, treatment is troublesome, so among the causes of complications of biliary tract surgery, Mirizzi syndrome is an important one. Despite a considerable number of reports of Mirizzi syndrome, none have described the process of its development from simple cholecystolithiasis.

Here, we report a rare case of Mirizzi syndrome with cholecystobiliary fistula, in which we were able to observe the process of its development from asymptomatic cholecystolithiasis over a course of 4 years.

## 2. Case Presentation

**Figure 1:**
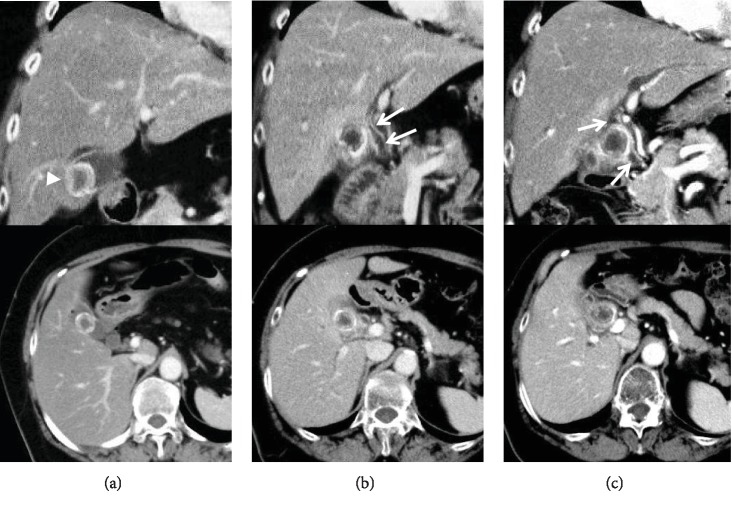
Abdominal CT scan (axial and coronal views): (a) 4 years ago, (b) 6 months ago, and (c) 2 months ago. The CT scan shows a 1.9 cm radiopaque stone (arrowhead in (a)). The gallbladder stone was compressing the common hepatic duct (arrows in (b)). The gallbladder stone had progressively compressed the common hepatic duct (arrows in (c)) in comparison with the situation 6 months previously.

A 68-year-old woman presented at our hospital with right upper quadrant pain. She had been diagnosed as having asymptomatic cholecystolithiasis 4 years previously. A diagnostic abdominal computed tomography (CT) scan had revealed a 1.9 cm radiopaque stone (Figure 1(a)), and thereafter, the patient was monitored by imaging alone once a year. The patient had shown neither symptoms nor changes on imaging until 6 months before the present admission, when CT revealed that the gallbladder stone was compressing the common hepatic duct, although the patient remained asymptomatic (Figure 1(b)). Subsequently, even though no symptoms were evident and liver function remained normal, abdominal CT conducted 2 months before the present admission showed progressive compression of the common hepatic duct by the gallbladder stone (Figure 1(c)).

**Figure 2:**
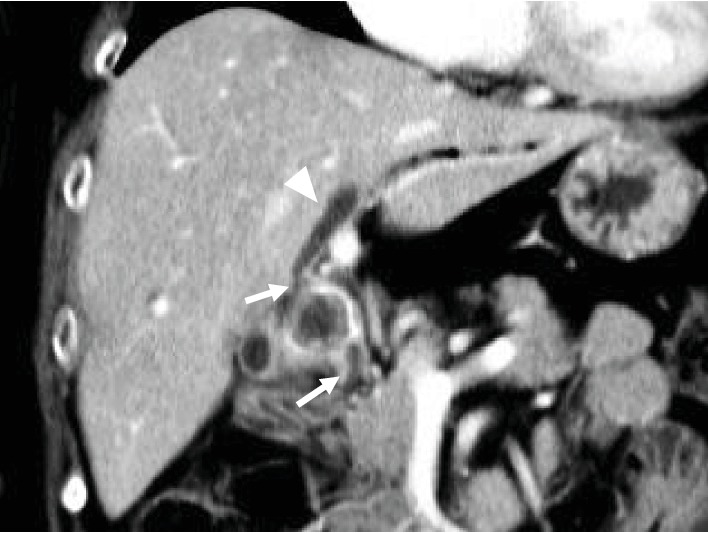
Abdominal CT scan on admission. The CT scan revealed that the gallbladder stone was obstructing the common bile duct (arrows) with dilatation of the intrahepatic duct (arrowhead).

**Figure 3:**
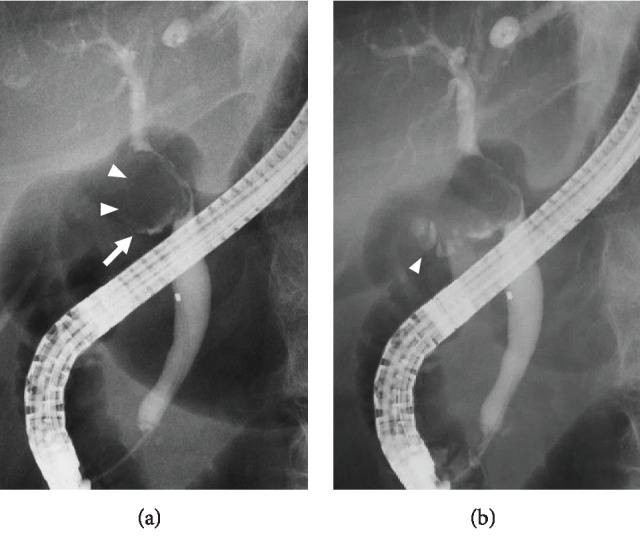
ERCP on admission. ERCP revealed a round filling defect of the bile duct (arrowheads in (a)) and inflow of contrast medium along the impacted gallstone directly (arrow in (a)). Subsequently, it further showed the naïve cystic duct which was separated from the filling defect (arrowhead in (b)).

**Figure 4:**
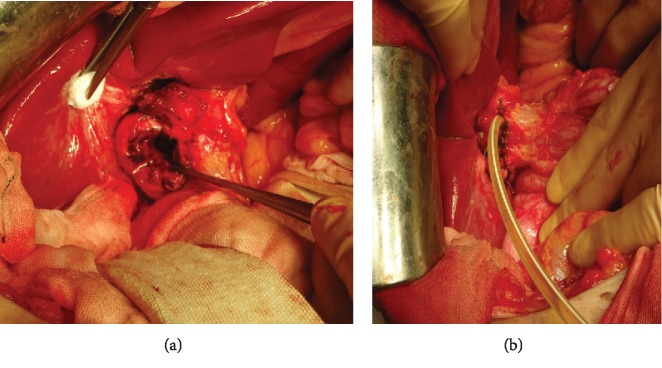
Intraoperative view. After removing the gallstone, a large cholecystobiliary fistula was found (a). This was repaired using the cuff of the gallbladder for fistula closure and distal placement of a T tube (b).

On admission, the patient's laboratory findings suggested abnormal liver function with elevated levels of alanine aminotransferase (320 U/L), aspartate aminotransferase (275 U/L), and total bilirubin (0.6 mg/dL). Abdominal CT showed that the gallbladder stone was obstructing the common bile duct with dilatation of the intrahepatic duct (Figure 2). Based on these findings, we diagnosed the patient as having Mirizzi syndrome and cholangitis. ERCP revealed a round filling defect of the bile duct and inflow of contrast medium along the impacted gallstone directly (Figure 3(a)). Subsequently, the naïve cystic duct was also identified; it separated from the filling defect (Figure 3(b)). These findings allowed preoperative diagnosis of cholecystobiliary fistula, and accordingly, the patient was categorized as having Mirizzi syndrome type III, based on the Csendes classification [2]. Liver function and the inflammation status improved rapidly after placement of a plastic biliary stent, and surgery was performed one month after ERCP. A subcostal incision revealed that the gallbladder had consolidated and hardened; subtotal cholecystectomy was performed, leaving a flap of the gallbladder wall to repair the bile duct. Upon opening of the gallbladder, a large gallstone was removed, revealing a cholecystobiliary fistula involving up to two-thirds of the circumference of the common bile duct (Figure 4(a)). The fistula was repaired using the cuff of the gallbladder for closure and distal placement of a T tube (Figure 4(b)). The postoperative course was uneventful, and the patient was discharged on day 17 after surgery.

## 3. Discussion

In 1982, McSherry et al. classified Mirizzi syndrome into two types, based on ERCP findings: type I involves external compression of the common hepatic duct by a large stone impacted within the cystic duct, or the Hartman pouch, whereas in type II, a cholecystocholedochal fistula is present, caused by a calculus that has eroded partly or completely into the common duct [3]. In 1989, Csendes and colleagues proposed subclassification of cholecystobiliary fistula (McSherry type II) into three categories (types II-IV) according to size: in type II lesions, there is a cholecystobiliary fistula with erosion of less than one-third of the circumference of the bile duct; in type III lesions, the fistula involves up to two-thirds of the duct circumference; and in type IV lesions, there is complete destruction of the bile duct [2]. The present patient was categorized as having Mirizzi syndrome type III, based on the ERCP findings.

Cholecystobiliary fistula has been explained by two mechanisms [4]. The first mechanism proposes that the impacted gallstone and its secondary inflammatory process will lead to complete obliteration of the cystic duct; the impacted gallstone seeking to pass into the bile duct will lead to the development of a pressure ulcer that will ultimately erode the gallbladder wall and bile duct wall, forming a communication between the two lumina. The second mechanism proposes that the impacted gallstone at the gallbladder infundibulum progressively dilates the cystic duct, leading to shortening, contraction, and fibrosis of this duct and finally forming a large communication between the gallbladder and bile duct. This type may, in fact, be a pseudofistula, where a large stone has eroded its way through the cystic duct. In the present case, the cholecystobiliary fistula was considered to have developed through the first of the above two mechanisms, because the image of the native cystic duct had been displayed on imaging previously. Here, we were able to use imaging to observe the progression of Mirizzi syndrome including cholecystobiliary fistula. From this viewpoint, the present case was extremely rare, as even though the syndrome developed rapidly, the etiological factors associated with it could not be clarified. In fact, when CT revealed that the gallbladder stone was compressing the common hepatic duct, we considered surgery to prevent the development of this syndrome. However, we were unable to plan surgery at that time because the patient had been admitted to another hospital due to right upper limb fracture.

With regard to treatment for Mirizzi syndrome, Beltrán has reported that ERCP has associated morbidity and mortality, and therefore, its risks must be weighed against its benefits [4]. Furthermore, most cholecystobiliary fistulas are diagnosed during surgery and not during preoperative studies. In this case, we considered that preoperative ERCP was indicated for relief of the severe cholangitis, and this allowed us to identify the cholecystobiliary fistula preoperatively. Different surgical strategies are used to treat Mirizzi syndrome in accordance with the Csendes classification [4, 5]. If the fistula is small and has eroded less than one-third of the circumference of the common bile duct, the defect can be sutured with fine absorbable sutures and a T tube can be placed distal to the fistula for 1 or 2 months. If the defect is larger, a cuff of the gallbladder is used for fistula closure and a T tube is placed distally.

Currently, laparoscopic cholecystectomy for Mirizzi syndrome is considered controversial and technically challenging, placing the patient at a probably unnecessarily increased risk of bile duct injury; as a consequence, laparoscopic cholecystectomy for Mirizzi syndrome cannot currently be recommended as a standard procedure [4, 6]. In the present case, we performed open surgery as the Csendes classification suggested that the large fistula justified this approach. A good outcome was obtained, and the postoperative course was uneventful.

To our knowledge, no previous reports have described the developmental process of Mirizzi syndrome from asymptomatic cholecystolithiasis. This is the first case report to use imaging to observe the progression of Mirizzi syndrome including cholecystobiliary fistula.
